# Auriculatone Sulfate Effectively Protects Mice Against Acetaminophen-Induced Liver Injury

**DOI:** 10.3390/molecules24203642

**Published:** 2019-10-09

**Authors:** Liangcai Lin, Huanyu Guan, Rui Li, Xiangming Liao, Feifei Zhao, Min Wang, Jing Li, Guobo Xu, Xun He, Jinjuan Zhang, Yongjun Li, Yonglin Wang, Meng Zhou, Shanggao Liao

**Affiliations:** 1 State Key Laboratory of Functions and Applications of Medicinal Plants & School of Pharmacy, Guizhou Medical University, Guizhou 550025, China; llcwx321@163.com (L.L.); guanhuanyu630@163.com (H.G.); lirui_318@163.com (R.L.); stanley_liaoxm@163.com (X.L.); zffei0225@sina.com (F.Z.); Lizhudele365@sina.com (M.W.); nigel_lijing@163.com (J.L.); xguobo@163.com (G.X.); 2 Engineering Research Center for the Development and Application of Ethnic Medicine and TCM (Ministry of Education), Guizhou Medical University, Guizhou 550004, China; liyongjun026@126.com (Y.L.); gywyl@gmc.edu.cn (Y.W.); 3 Guizhou Provincial Key Laboratory of Pharmaceutics, Guizhou 550004, China; hexun224@gmc.edu.cn; 4 School of Medicine and Health Management, Guizhou Medical University, Guizhou 550025, China; 5 School of Basic Medical Sciences, Guizhou Medical University, Guizhou 550025, China; zjj216@163.com

**Keywords:** Aacetaminophen-induced hepatotoxicity, hepatoprotective effect, auriculatone sulfate, oxidative stress, inflammation, CYPs inhibition

## Abstract

Acetaminophen (APAP) overdose is very common worldwide and has been widely recognized as the leading cause of drug-induced liver injury in the Western world. In our previous investigation, auriculatone, a natural product firstly obtained from *Aster auriculatus*, has demonstrated a potent protective effect against APAP-induced hepatotoxicity in HL-7702 cells. However, the poor water solubility and low bioavailability restrict its application. Auriculatone sulfate (AS) is a sulfated derivative of auriculatone with highly improved water-solubility. Hepatoprotective effects against APAP-induced liver injury (AILI) showed that intragastric pretreatment with AS at 50 mg/kg almost completely prevented mice against APAP-induced increases of serum alanine aminotransferase, aspartate aminotransferase, lactate dehydrogenase and ATPase. Histological results showed that AS could protect the liver tissue damage. In addition, AS pretreatment not only significantly retained hepatic malondialdehyde and the activities of glutathione, superoxide dismutase, and glutathione peroxidase at normal levels, but also markedly suppressed the increase of pro-inflammatory cytokines TNF-α, IL-1β, and IL-6 levels in mouse liver caused by overdose APAP. Immunohistochemical analysis showed that AS obviously attenuated the expression of CD45 and HNE in liver tissue. Further mechanisms of action investigation showed that inhibition of cytochrome P450 3A11 (CYP 3A11) and CYP2E1 enzymatic activities (but not that of CYP1A2) was responsible for APAP bioactivation. In conclusion, AS showed a hepatoprotective effect against AILI through alleviating oxidative stress and inflammation and inhibiting CYP-mediated APAP bioactivation. It may be an effective hepatoprotective agent for AILI and other forms of human liver disease.

## 1. Introduction

Drug-induced liver injury (DILI) has been the most common cause of acute liver failure in recent years [1]. Acetaminophen (APAP) is a safe antipyretic and analgesic drug at therapeutic doses. However, APAP overdose-induced liver injury is not uncommon worldwide and has been recognized as the leading cause of DILI in Great Britain and in the United States [2]. At therapeutic doses, APAP is metabolized mainly to sulfated and glucuronidated conjugates to be further excreted in the urine [3], while a minor portion of APAP is metabolized by cytochrome P450 enzymes (CYPs) (e.g., CYP1A2, CYP2E1, and CYP3A4) into an electrophilic and toxic form, *N*-acetyl-p-benzoquinone-imine (NAPQI) [4]. NAPQI can be readily detoxified by conjugation into glutathione (GSH). However, an overdose of APAP can saturate its glucuronidation and sulfation, resulting in accumulation of NAPQI, excessive conjugation of which to GSH afterwards leads to depletion of the hepatic GSH pool. Subsequently, extra NAPQI covalently binds to hepatocyte proteins (mitochondrial proteins in particular), gives rise to mitochondrial dysfunction, and ultimately leads to liver injury and necrosis [5,6,7].

**Figure 1:**
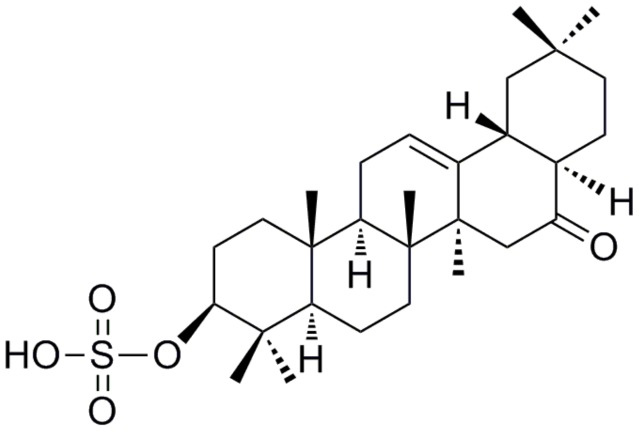
The chemical structure of auriculatone sulfate.

At present, *N*-acetyl cysteine (NAC), a substrate for GSH synthesis that alleviates APAP hepatotoxicity by elevating the hepatic GSH pool [8,9], is still one of the rare drugs used for the treatment of APAP-induced liver injury (AILI). However, NAC has side effects like nausea, vomiting, and rare fatal anaphylactoid reactions which might occur [10,11]. Natural products have been the main source of hepatoprotective agents. Silymarin, glycyrrhizic acid, and oleanolic acid are among the well-known hepatoprotective agents that are widely used in clinic. However, the protective effects of these agents are far from satisfactory and more effective hepatoprotective agents are required [12]. Auriculatone, a norpentacyclic triterpenoid compound, was firstly obtained as a natural product from *Aster auriculatus* [13]. In our previous investigation, auriculatone, which was obtained as an impurity from a clinically used hepatoprotective oleanolic acid tablet, has demonstrated a potent protective effect against APAP-induced hepatotoxicity in HL-7702 cells [14]. However, due to poor water solubility and low bioavailability, its practical application has been restricted. Efforts toward the development of auriculatone and its derivatives for clinical use have led to the discovery of a more powerful hepatoprotective agent: auriculatone sulfate (AS) (Figure 1). AS is a sulfated derivative of auriculatone with highly improved water-solubility. The present investigation showed that AS could effectively protect mice against AILI. In addition, a preliminary study of its mechanism of action was also reported.

## 2. Results

### 2.1. Protection of the Liver by AS against the APAP-Induced Injury in Mice

**Figure 2:**
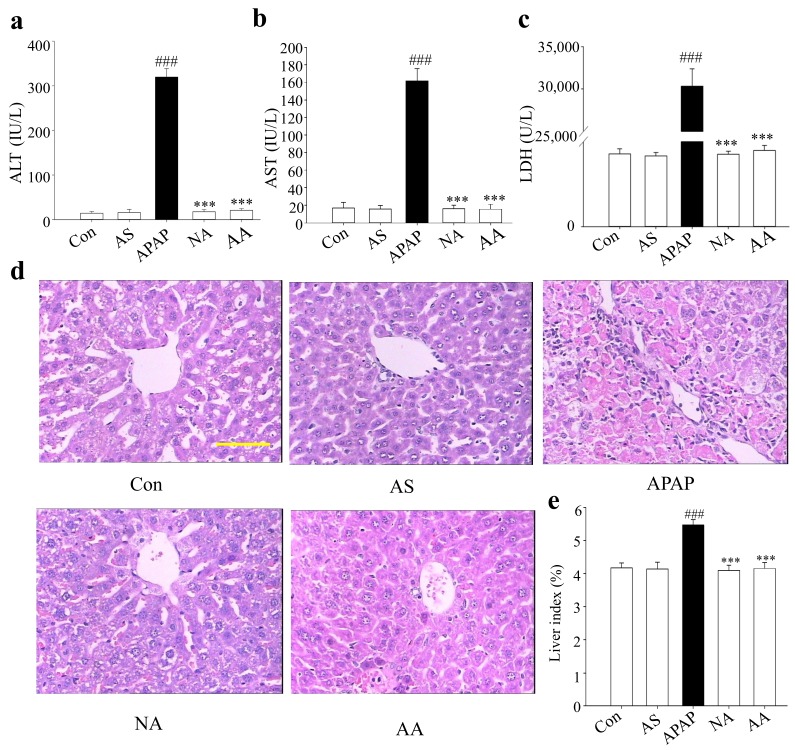
Pretreatment of Auriculatone sulfate (AS) prevents mice from Acetaminophen (APAP)-induced acute liver injury. Control group (Con), AS (50 mg/kg)-treated group (AS), APAP (300 mg/kg)-treated group (APAP), AS (50 mg/kg)/APAP (300 mg/kg)-treated group (AA), *N*-acetyl cysteine (NAC) (1000 mg/kg)/APAP (300 mg/kg)-treated group (NA). (**a**) Serum alanine aminotransferase (ALT) levels. (**b**) Serum aspartate aminotransferase (AST) levels. (**c**) Serum lactate dehydrogenase (LDH) levels. (**d**) Representative histological staining (H&E) of liver tissue (400 ×), scale bar: 10 μm. (**e**) Liver index. Data are expressed as means ± SD (*n* = 10). ^###^ *p* < 0.001 compared to Con group; ^***^ *p* < 0.001 compared to APAP group.

To evaluate the hepatoprotective effect of AS, serum biochemical parameters including alanine aminotransferase (ALT), aspartate aminotransferase (AST), and lactate dehydrogenase (LDH) were assayed. The effects of AS on the change of serum biochemical parameters induced by APAP are shown in Figure 2a–c. Compared to the control group, serum ALT, AST, and LDH levels in APAP-treated mice were increased significantly (*p* < 0.001) from 14.39 ± 3.92 U/L, 16.59 ± 6.51 U/L, and 513.63 ± 35.14 U/L to 319.56 ± 19.09 U/L, 161.72 U/L, and 30357.54 ± 2018.61 U/L, respectively, in the APAP group. The results showed that an overdose of 300 mg/kg APAP could induce severe liver injury. However, as indicated in the AA and NA groups, AS and NAC treatment significantly prevented the increases of serum enzyme levels (*p* < 0.001). Intragastric administration of 50 mg/kg AS prior to APAP treatment significantly decreased the serum ALT, AST, and LDH levels to 20.81 ± 3.80 U/L, 15.36 ± 5.68 U/L and 536.54 ± 35.27 U/L, respectively. The potency was very close to that observed for intravenous injection of NAC at 1000 mg/kg 1 h after APAP administration. The results clearly demonstrated that AS could almost completely protect against AILI.

### 2.2. Effects of AS on Liver Histopathology

As indicated in Figure 2d, when compared with the normal control, APAP-treated mice exhibited damage of liver tissue structure, with large areas of centrilobular hepatocellular necrosis, lymphocyte infiltration extending into the hepatic lobule, and signs of inflammation, whereas mice post-treated with 1000 mg/kg NAC and pre-treated with 50 mg/kg AS displayed, similar to the normal control group, normal liver tissue structure, clear hepatic lobules, mild centrilobular hepatocellular necrosis, and normal lymphocyte infiltration. Moreover, compared to the APAP group, the liver indexes of the NA group and AA group were significantly decreased (*p* < 0.001) (Figure 2e).

### 2.3. AS Inhibits APAP-Induced Hepatic Mitochondrial Injury

**Figure 3:**
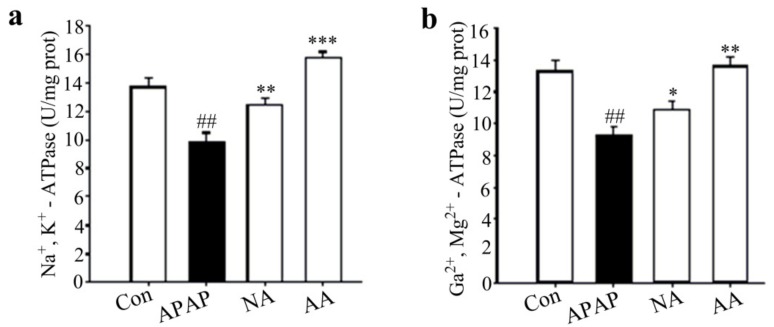
Effects of AS pretreatment on mitochondrial Na^+^, K^+^-ATPase and Ca^2+^, Mg^2+^-ATPase during APAP-induced hepatotoxicity. The activities of Hepatic Na^+^, K^+^-ATPase (**a**) and Hepatic Ca^2+^, Mg^2+^-ATPase (**b**) were expressed in U/mg protein (prot). Data are presented as means ± SD (*n* = 10). ^##^ *p* < 0.01 compared to the Con group; ^*^p < 0.05, ^**^p < 0.01, ^***^p < 0.001 compared to the APAP group.

As shown in Figure 3, APAP treatment significantly (*p* < 0.01) decreased the activities of mitochondrial Ca^2+^, Mg^2+^-ATPase and Na^+^, and K^+^-ATPase in mice, indicating the presence of mitochondrial dysfunction. However, pre-treatment with AS or post-treatment with NAC significantly (*p* < 0.05) ameliorated the mitochondrial dysfunction by mitochondrial enzyme activities. It is apparent that AS is much stronger than NAC as the values for the two enzymatic activities in the AA group have been elevated to levels not significantly different from those of the Con group (*p* > 0.5).

### 2.4. Effects of AS on TNF-α, IL-1β, and IL-6 Levels in the Liver

**Figure 4:**
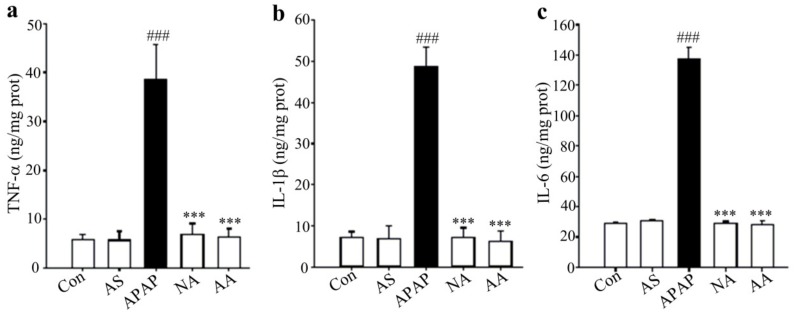
Effects of AS on proinflammatory cytokines. (**a**) Hepatic TNF-α levels. (**b**) Hepatic IL-1β levels. (**c**) Hepatic IL-6 levels. Data are expressed as means ± SD (*n* = 10). ^###^*p* < 0.001 compared to the Con group; ^***^p < 0.001 compared to the APAP group.

Cytokines are protein mediators of inflammation. As shown in Figure 4, the hepatic levels of TNF-α, IL-1β, and IL-6 were significantly increased in APAP-treated mice (*p* < 0.001). However, AS treatment significantly suppressed the release of the three cytokines (*p* <0.001). The results suggested that reduction of inflammatory mediators in the liver was involved in the hepatoprotective effect of AS against AILI.

### 2.5. Effects of AS on MDA, SOD, GSH and GSH-PX Levels of Liver Tissues

**Figure 5:**
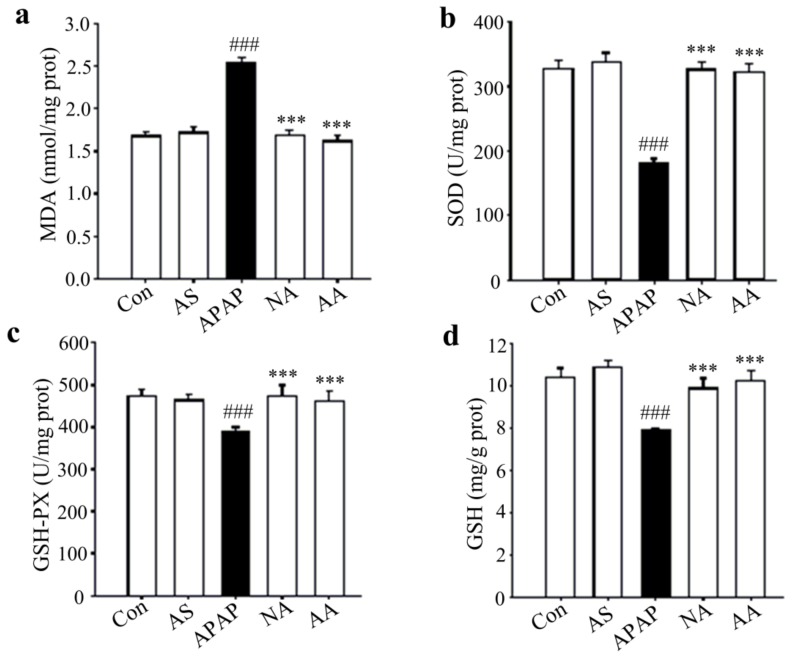
AS decreases hepatic oxidative stress in APAP-induced hepatotoxicity mice. (**a**) Hepatic MDA levels. (**b**) Hepatic SOD activities. (**c**) Hepatic GSH-PX activities. (**d**) Hepatic GSH levels. Data are expressed as means ± SD (*n* = 10). ^###^ *p* < 0.001 compared to the Con group; ^***^p < 0.001 compared to the APAP group.

To understand the mechanisms of AS for its protective effect against acute APAP-induced liver injury, the levels of antioxidant enzymes superoxide dismutase (SOD), glutathione peroxidase (GSH-PX), glutathione (GSH), and malondialdehyde (MDA) in liver tissues were determined. As shown in Figure 5, APAP treatment markedly elevated MDA production and reduced SOD, GSH, and GSH-PX contents in the liver. The MDA levels were significantly increased by 1.51 fold in the APAP group as compared to those in the control group, while oral administration of 50 mg/kg AS prior to APAP treatment almost completely inhibited the APAP-induced elevation (*p* < 0.001) (Figure 5a). Meanwhile, SOD, GSH, and GSH-PX activities were almost elevated to the normal values (*p* < 0.001, Figure 5b–d). Similar effects were also observed for NAC treatment. The results demonstrated that AS protected the liver from APAP-induced damage via prevention of its oxidative injury.

### 2.6. Immunohistochemical Examination

**Figure 6:**
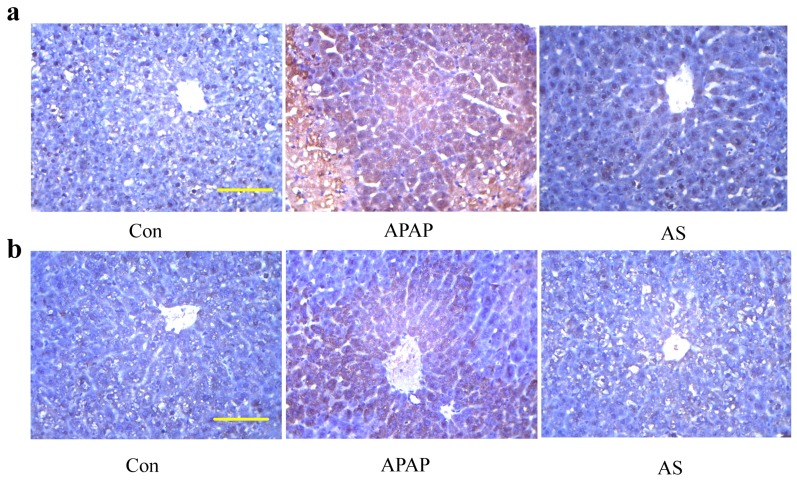
AS pretreatment inhibited the increases of CD45 and HNE-adducts caused by overdose APAP in mice. Analysis with immunohistochemical staining of effects of AS on the expression of CD45 (**a**) and HNE-adducts (**b**). Slides were observed under 400 × magnification,scale bar: 10 μm.

As indicated in Figure 5, although very limited expression of CD45 was observed in the liver cytoplasm of the control mice, strongly positive expression of CD45 was detected in that of the APAP-injured mice (Figure 6a). However, inflammation-related CD45 expression initiated by APAP was clearly inhibited by AS treatment. Further immunohistochemical investigation on the lipid peroxidation marker HNE showed that HNE showed virtually no staining of HNE-modified proteins in the control group, but significant expression of HNE-adducts was observed in the APAP group, whereas AS treatment obviously decreased the HNE expression to almost the normal level of the control group (Figure 6b).

### 2.7. Effects of AS on the Expressions and Activities of CYPs

**Figure 7:**
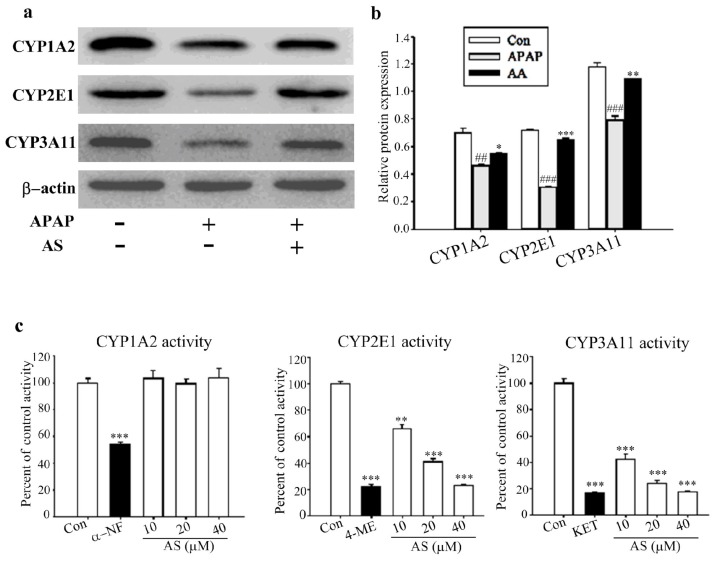
Effects of AS on the expressions and activities of CYP1A2, CYP2E1 and CYP3A11. Western-blot analysis (**a**) and the relative density ratio (**b**) of CYP1A2, CYP2E1, and CYP3A11 in liver tissues. (**c**) AS possesses significant inhibitory effects on the activities of CYP1A2, CYP2E1 and CYP3A11. Inhibitory effects of AS on CYPs activity were measured by LC-MS/MS-based cocktail incubation approach in vivo. Data are expressed as means ± SD (*n* = 3). ^##^*p* < 0.01, ^###^*p* < 0.001 compared to the Con group; ^*^p < 0.05, ^**^p < 0.01, ^***^p < 0.001 compared to the APAP group.

CYPs play a key role in the development of APAP-induced hepatotoxicity through APAP metabolic activation and formation of the toxic reactive metabolite NAPQI. In the current investigation, the effects of AS on the expression and activities of CYPs were evaluated. Compared to the control group, the expression levels of CYP1A2, CYP2E1 and CYP3A11 in the APAP group were obviously decreased, but the reductions were significantly inhibited by AS pretreatment (Figure 7a,b). In the enzyme activity assays, remarkable inhibitory effects on the original activities of CYP2E1 and CYP3A11 in the mouse liver microsomes were observed for AS, with IC_50_ of 6.5 and 16.0 μM, respectively (Figure 7c). However, the activity of CYP1A2 was not influenced by AS (Figure 7c).

## 3. Discussion

ALT and AST are the most sensitive biomarker enzymes used to assess liver function. Liver dysfunction and injury can cause remarkable increases of serum ALT and AST levels. In line with previous studies [15], the serum levels of ALT and AST were significantly elevated after intraperitoneal administration of APAP at 300 mg/kg. Besides, a noteworthy increase of the LDH level in serum was also observed for APAP overdose, suggesting damage of the plasma membranes [16]. The observation of significant decreases of ALT, AST, and LDH in the AS pretreatment group to the levels close to those of the control group suggested that AS was very powerful as a hepatoprotective agent and its intragastric administration (50 mg/kg) could almost completely protect mice against AILI. The potency was comparable to that of NAC intravenously injected at 1000 mg/kg 1 h after APAP administration. Histological study further confirmed the conclusion.

The hepatocyte proteins (e.g., ATP synthetase) covalently binding with NAPQI are primarily localized in the mitochondria [17,18]. In cases where binding occurs, the activity of ATP synthetase will be inhibited, leading to mitochondrial energy supply dysfunctions in the hepatocytes [19,20,21]. Our results showed that the decreases of mitochondrial Ca^2+^, Mg^2+^-ATPase, and Na^+^, K^+^-ATPase in APAP hepatotoxicity were completely inhibited by administration of 50 mg/kg AS. It was apparent that APAP-induced mitochondrial dysfunction was totally prevented by AS.

After an administration of overdose APAP, abundant inflammation with significantly increased levels of pro-inflammatory cytokines TNF-α, IL-1β and IL-6 occurs in the liver tissue, which subsequently enhances liver tissue injury [22,23,24]. Inhibition of the releases of these cytokines was found to be an effective strategy to reduce APAP hepatotoxicity [25]. Besides, since cytoplasm was the main site of expression of the inflammation marker CD45, positive expression of cytoplasmic CD45 was associated with AILI [26]. Remarkable inhibition of pro-inflammatory cytokine (TNF-α, IL-1β and IL-6) as well as cytoplasmic CD45 expressions by AS pretreatment suggested that inhibition of liver inflammation was involved in the hepatoprotective effect of AS against APAP-induced hepatotoxicity.

APAP was metabolized, by cytochrome P450 enzymes, to the toxic two-electron oxidation product *N*-acetyl-p-benzoquinone-imine (NAPQI) [27,28]. By forming a NAPQI-GSH conjugate, NAPQI is detoxified. After an overdose of APAP, hepatic GSH is rapidly depleted by as much as 90% [29]. Subsequently, extra NAPQI binds to cellular protein and liver oxidative damage occurs [30]. MDA is a particular biomarker of tissue oxidative damage, and over expression of MDA is an indication of the breakdown of the antioxidant system against oxidative stress [31,32]. AS significantly inhibited the increase of MDA induced by overdose APAP and maintained the antioxidative system parameters (e.g., SOD, GSH, and GSH-PX) to their normal levels. The potency was very close to that of NAC intravenously post-injected at 1000 mg/kg. HNE is one of the major cytotoxic end products of lipid peroxidation [33,34], effective inhibition of the APAP-induced expression of HNE by AS indicates that the protective effect of AS against APAP hepatotoxicity was also associated with its prevention of the hepatic antioxidative defense system.

Metabolic activation of APAP is a key initiating event in APAP hepatotoxicity. CYPs such as CYP3A11, CYP2E1, and CYP1A2 were reported to be involved in the activation of APAP in mice [35,36,37], with CYP3A11 and CYP2E1 being the most important two CYPs responsible for the formation of NAPQI-GSH [38]. Although AS strongly restored the expressions of the three CYP enzymes, only inhibition of CYP3A11 and CYP2E1 enzymatic activities (but not that of CYP1A2) was observed for APAP bioactivation. This observation was consistent with the previous report stating that the enzymatic activities of CYPs were more important than their expression levels for APAP hepatotoxicity [39]. The results were also in agreement with those reported for auriculatone against APAP-induced hepatotoxicity in HL-7702 cells, where inhibition of CYP3A4 (CYP3A11 in mice) activity was shown to be one mechanism responsible for its hepatoprotective activity [14].

APAP hepatotoxicity has become one of the most serious health problems worldwide. Overdose of APAP may cause a wide range of adverse effects [40,41]. Although quite a number of compounds (e.g., silymarin, glycyrrhizic acid and oleanolic acid) were shown to possess hepatoprotective effect against APAP-induced hepatotoxicity [42], their hepatoprotective effects and mechanisms of action might not be satisfactory enough, and administration of NAC is still one of the best clinical therapies for APAP-induced liver toxicity. Our study demonstrated that AS almost completely prevented APAP-induced liver injury as NAC in every aspect, and the hepatoprotection is associated with its retaining expression of CYP enzymes and inhibition of the activities of CYP2E1 and CYP3A11 to decrease APAP bioactivation. AS might be an effective hepatoprotective agent for AILI and other forms of human liver disease.

## 4. Materials and Methods

### 4.1. Chemicals and Reagents

Auriculatone sulfate (AS) was obtained as a derivative of auriculatone in our lab and was structurally determined by NMR and MS data. NADPH tetrasodium salt, chlorzoxazone (CHL), nifedipine (NIF), phenacetin (PHE), 4-methylpyrazole (4-ME), α-naphthoflavone (α-NF), and ketoconazole (KET) were obtained from J&K Scientific (Beijing, China). Acetaminophen (APAP) and *N*-acetyl-cysteine (NAC) were obtained from Yuanye Biology (Shanghai, China). 6-Hydroxychlorzoxazone (OHCHL) and dehydronifedipine (DNIF) were obtained from TLC Pharmaceutical standards (Aurora, Ontario, Canada). CYP2E1, CYP1A2, CD-45, HNE, and β-actin antibodies were from Abcam (Cambridge, UK). CYP3A11 antibody was obtained Santa Cruz biotechnology (Santa Cruz, CA, USA). The secondary antibody was obtained from Abcam (Cambridge, UK). Mouse liver microsomes were obtained from Wuhan PrimeTox Bio-medical Technology Co., LTD. (Wuhan, China). Kits for assaying levels of alanine aminotransferase (ALT), aminotransferase (AST), lactate dehydrogenase (LDH), mitochondrial ATPase, malondialdehyde (MDA), glutathione (GSH), superoxide dismutase (SOD), glutathione (GSH), and glutathione peroxidase (GSH-PX), tumor necrosis factor α (TNF-α), interleukin 6 (IL-6), and interleukin 1β (IL-1β) were purchased from Nanjing Jiancheng Biology Engineering Institute (Nanjing, China). Other reagents used in the experiment were all of analytical grade.

### 4.2. Animals and Treatments

Kunming strain mice (6–8 weeks old), weighting 18–22 g, were provided by the Laboratory Animal Center of Guizhou Medical University. Mice were kept, with a 12 h light/dark cycle, in an environmentally controlled room with temperature of 22–24 °C and relative humidity of 55%–60%. The mice were acclimatized 1 week prior to the experiment. Food and water were freely available. All animal protocols were approved by the Ethics Committee on the Care and Use of Laboratory Animals of Guizhou Medical University.

All the mice were randomly separated into five groups, with 10 mice in each group: Control group (Con), AS (50 mg/kg)-treated group (AS), APAP (300 mg/kg)-treated group (APAP), AS (50 mg/kg)/APAP (300 mg/kg)-treated group (AA), NAC (1000 mg/kg)/APAP (300 mg/kg)-treated group (NA). Mice were administered AS (50 mg/kg, dissolved in 3% Tween-80 saline) (for groups AS, AA, and NA) or 3% Tween-80 saline (for groups Con, APAP, and NA) by gavage for 3 consecutive days. All mice were fasted for 15 h before APAP administration. 2 h after the last dose of AS, all mice except those in the control group were injected with APAP intraperitoneally at 0.2mL/10g. APAP was dissolved in saline (40 °C) and administered by intraperitoneal injection at 300 mg/kg (body weight). 1 h after the dose of APAP, NA group mice were administered NAC (1000mg/kg, dissolved in saline) by intravenous injection (0.1mL/10g), while mice in the other groups were given saline intravenously. All animals were killed at 24 h after APAP treatment. Blood samples were collected and the serum was separated by centrifugation at 3000 rpm, 4 °C for 10 min, and then stored at −20 °C prior to AST, ALT and LDH analysis. After the mice were sacrificed, their liver tissues were promptly dissected out, then washed with ice-cold saline, blotted with filter papers to remove the surface water, and weighed for liver index calculation. Liver index was estimated from the ratio of liver weight to body weight. The liver index was calculated according to the formula: (mice liver weight/mice weight) × 100%. Finally, the liver was divided into two parts. One was immediately fixed in 10% buffered formalin for pathological examination, and the remaining tissues were flash frozen in liquid nitrogen and stored at −80 °C for further use.

### 4.3. Assays of Serum ALT, AST and LDH

Serum samples were prepared as in Section 4.2. Levels of serum alanine aminotransferase (ALT), aspartate aminotransferase (AST), and lactate dehydrogenase (LDH) were measured spectrophotometrically based on the instructions given in the assay kits (Nanjing Jiancheng Institute of Biotechnology, China).

### 4.4. Determinations of Hepatic MDA, SOD, GSH and GSH-PX

Liver tissues were thawed and homogenized in ice-cold saline and the homogenate was centrifuged at 4 °C, 3000 rpm for 10 min. Then the supernatants were assayed for malondialdehyde (MDA), superoxide dismutase (SOD), GSH and glutathione peroxidase (GSH-PX) activities using the commercially available assay kits as per manufacturer’s instruction (Nanjing Jiancheng Institute of Biotechnology, China). Protein contents of liver homogenates were determined based on the instructions of a BCA protein assay kit (Nanjing Jiancheng Institute of Biotechnology, China).

### 4.5. Determination of Cytokines

Liver homogenates were prepared as described in Section 4.4. Levels of hepatic TNF-α, IL-1β and IL-6 were measured using commercial ELISA kits according to the manufacturer’s instructions (Nanjing Jiancheng Institute of Biotechnology, China).

### 4.6. Preparation of Mitochondria and Measurement of Mitochondrial ATPase Activity

The method for separating mitochondria from the liver tissue was conducted according to Tissue Mitochondria Isolation Kit (Nanjing Jiancheng Bioengineering Institute, China). Briefly, 1 g of the rinsed and filter paper-dried liver from the sacrificed mouse was homogenized with 9 mL of ice-cold separating reagent (100 mL water with 75 mmol/L sucrose, 225 mmol/L mannitol, 0.05 mmol/L EDTA, and 10 mmol/L Tris, adjusted with 1 mol/L HCl to pH 7.4). The supernatant obtained was first centrifuged at 600 rpm for 10 min at 4 °C. The supernatant obtained was further centrifuged at 88,000 rpm for 10 min at 4 °C. The sediment was washed three times with precooled (4°C) separating reagent to give purified liver mitochondria, resuspension of which in 800 μL of separating reagent gave a mitochondrial suspension which was then stored at −80 °C. Protein concentrations were determined according to the BCA protein assay kit.

For measurement of the liver mitochondrial ATPase activity, the liver mitochondria were diluted 8 times with the diluting reagent given in the kit. The OD value of each sample at 660 nm was determined by a UV-Vis spectrophotometer according to the procedure in the instructions of the ATPase kit. Then, the contents of Na^+^, K^+^-ATPase and Ca^2+^, and Mg^2+^-ATPase in each sample were calculated according to the formula given in the kit.

### 4.7. Histological Studies

Liver tissues were fixed in 10% buffered formalin, embedded in paraffin, sectioned (5 μm), stained with hematoxylin and eosin (H&E) and examined under a light microscope (Olympus BX51, Tokyo, Japan).

### 4.8. Immunohistochemical Staining

Livers were taken and fixed in 10% buffered formation, embedded in paraffin and cut into 10 μm sections. After blockade of inner peroxidase, sections were incubated sequentially with the anti-CD45 and HNE antibody. After five washes with PBS, the sections were incubated in the secondary antibody conjugated with peroxidase-labeled polymer, prior to colorization using diaminobenzidine reaction and counter stained with hematoxylin.

### 4.9. Measurement of Liver CYP Activity

CYP1A2, CYP2E1, and CYP3A11 are the main enzymes responsible for APAP transformation to NAPQI. The effects of AS on the activities of the CYPs were evaluated using an LC-MS/MS-based “cocktail” incubation approach as previously described, with slight modification [39,43]. Briefly, various concentrations of AS or positive inhibitors (α-NF at 0.2 μM for CYP1A2, 4-ME at 5 μM for CYP2E1, or KET at 1.1 mM for CYP3A11) and the mixed CYPs probe substrates (PHE for CYP1A2, CHL for CYP2E1, and NIF for CYP3A11) were “cocktail”-incubated with mouse liver microsomes. The metabolites (PAR/OHCHL/DNIF) of these CYPs probe substrates in all samples were determined using a previously developed LC-MS/MS method [44]. The IC_50_ values were calculated by Graph Prism 5 (GraphPad Software Inc., San Diego, CA, USA).

### 4.10. Western Blot Analysis

Western blot analysis was performed to analyze the expression of CYP1A2, CYP2E1 and CYP3A11 in liver tissue as reported [45]. Briefly, protein extracted from liver tissue was prepared using RIPA lysis buffer (Solarbio, Beijing, China) according to the manufacturer’s instructions. Protein concentrations were assayed following the manufacturer’s instructions for BCA protein assay. Each sample was normalized to 40 μg of the protein concentration. Protein extracts were separated in 10% sodium dodecyl sulfate polyacrylamide gel electrophoresis (SDS-PAGE) and electrophoretically transferred onto polyvinylidene fluoride membranes. After being blocked with 5% nonfat dry milk, the membranes were incubated with primary antibody overnight. The immunoblots were then incubated with a secondary antibody conjugated with horseradish peroxidase for 2 h at room temperature. The membranes were developed using an electrochemiluminescence (ECL) kit (Solarbio, Beijing, China) according to the manufacturer’s protocol and detected with a BOXChemiXL1.4 system (Biorad, Hercules, CA, USA).

### 4.11. Statistical Analysis

Data were expressed as mean ± standard deviation (SD). All data were statistically analyzed by one way analysis of variance (ANOVA) or *t* test using SPSS Statistics (version 13.0, IBM Corporation, Armonk, NY, USA). Significance was considered when *p* < 0.05. All data were assessed from at least three independent experiments.
